# Penetration and Displacement Behavior of N_2_ in Porous Interlayer Structures Containing Water/Salt Component by Molecular Dynamics Simulation

**DOI:** 10.3390/molecules26175168

**Published:** 2021-08-26

**Authors:** Zhibin Jiang, Liqiang Sima, Lisha Qi, Xiaoguang Wang, Jie Wang, Zhenpeng Leng, Tianpeng Zhao

**Affiliations:** 1 School of Geoscience and Technology, Southwest Petroleum University, Chengdu 610500, China; donghao15610029171@163.com; 2 Research Institute of Exploration and Development, PetroChina Xinjiang Oilfield Company, Karamay 834000, China; 15856956521@163.com (L.Q.); mengchen0628@163.com (X.W.); 17860739118@163.com (J.W.); 3 Institute of Mechanics, Chinese Academy of Sciences, Beijing 100190, China; lengsnupc@163.com; 4 Oleum Technologies (Beijing) Company Limited, Beijing 102206, China; xhl15621429029@163.com

**Keywords:** molecular dynamics simulation, penetration and displacement, porous interlayer structures

## Abstract

The penetration and displacement behavior of N_2_ molecules in porous interlayer structures containing a water/salt component with porosities of 4.29%, 4.73%, 5.17%, 7.22%, and 11.38% were explored using molecular dynamics simulations. The results demonstrated that the large porosity of the interlayer structures effectively enhanced the permeation and diffusion characteristics of N_2_. The water and salt in the interlayer structures were displaced during the injection of N_2_ in the porosity sequence of 4.29% < 4.73% < 5.17% < 7.22% < 11.38%. The high permeance of 7.12 × 10^−6^ indicated that the interlayer structures with a porosity of 11.38% have better movability. The strong interaction of approximately 15 kcal/mol between N_2_ and H_2_O had a positive effect on the diffusion of N_2_ and the displacement of H_2_O before it reached a stable equilibrium state. The distribution of N_2_ in porous interlayer structures and the relationship between the logarithm of permeability and breakthrough pressure were presented. This work highlighted the effects of porosity on the permeability and diffusion of N_2_/H_2_O in the interlayer, thus providing theoretical guidance for the development of petroleum resources.

## 1. Introduction

Oil has aroused growing attention as a crucial energy resource around the world [1,2,3]. Oilfield exploration technology is vigorously investigated to discover more oil resources [4,5,6]. The porous interlayer is one of the most important factors affecting the distribution of the remaining oil, which increases the heterogeneity of the reservoir [7], complicates the oil–water movement relationship [8], and disorders the distribution regularity of the remaining oil [9,10]. Previous studies have mainly focused on explaining the influence of the interlayer on oil–gas movement [11]. By exploring the effect of three types of interlayers on oil–water movement and the formation of rised water, Wang et al. [12] found that the oil layer can be divided into several connected or disconnected units and that it controls the movement of oil and water and ultimately affects the distribution of remaining oil. Luo et al. [13] discovered that the vertical migration interval of the oil and gas in the heterogeneous transport layer is hindered by the interlayer, thus complicating the migration path. Wu et al. [14] found that the interlayer restricts the vertical permeability of the reservoir. These results indicated that the interlayer has an important influence on oilfield exploration technology [15]. Considering the complexity of experimental conditions, theoretical calculation provides an effective way to explore the interlayer. The process of gases, including CO_2_ and N_2_, passing through the interlayer is the best method to define structural features. Fang et al. found that slug injection of CO_2_ was a promising technology for the enhancement of oil recovery in an MD simulation. However, few studies have focused on the injection of N_2_ into the interlayer [3], which could effectively open the channel for the migration of oil and gas and cause the remaining water/salt component to be the only substances in the interlayer. Therefore, it is vital to elucidate the effects of porosity on the interlayer for N_2_ penetration, H_2_O displacement behavior, and the diffusion mechanism.

In this work, we investigated the penetration and displacement behavior of N_2_ in interlayers containing a water/salt component and with porosities of 4.29%, 4.73%, 5.17%, 7.22%, and 11.38% using molecular dynamics (MD) simulations. First, porous SiO_2_ was built to simulate the interlayers. Subsequently, the pores of SiO_2_ were filled with a salt solution, followed by the displacement of N_2_ molecules under breakthrough pressure. Next, the diffusion process and mean squared displacement were calculated in consideration of the porosity and pressure effects. Then, the gas distribution and the permeability–pressure relationship were analyzed to show the microscopic mechanism of N_2_/water/salt passing through the interlayer. Our results highlight the potential impact of the interlayer on the gas diffusion process, providing theoretical guidance for oil exploitation.

## 2. Model and Method

The silica structure was selected as a typical mineral component [16]. The generation of the frameworks was conducted using a 10 × 10 × 10 supercell of silica structure with dimensions (xyz) of 71.60 Å × 71.60 Å × 71.60 Å. Considering that the remaining oil exists in interlayer structures with porosities of 4–8% [17], pores along the *z*-axis with porosities of 4.29%, 4.73%, 5.27%, 7.22%, and 11.38% were established in the center of the silica structures. These structures were tagged as P4.29%, P4.73%, P5.27%, P7.22%, and P11.38%. Furthermore, the silica structures used in this study were rigid and fixed, and the silica surface of the pore was hydroxylated and set at 9.6 nm^−2^ [17]. 

**Figure 1:**
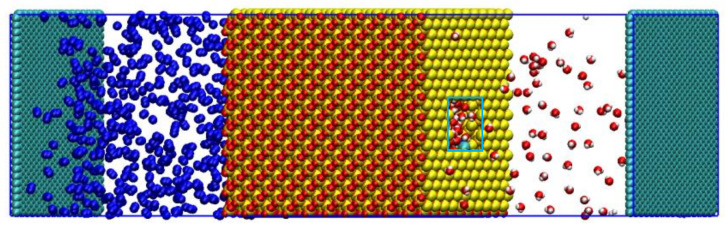
Initial configurations of N_2_ in the porous interlayer.

MD regards all matter as a particle system composed of atoms and molecules, and the motion of all particles complies with the laws of classical mechanics or quantum mechanics. The most important characteristic of MD is that it ignores the interactions of atoms between electrons and electrons and the electrons and the nucleus, only regarding the atom as a basic unit to calculate the energy of the whole system and thus greatly improving the computational efficiency, making the MD method applicable to larger computing systems. Therefore, the MD simulation was adopted to complete these calculations. A saline solution with an H_2_O to salt molar ratio of 1000:1 [18] was uniformly distributed in the hydroxylated silica pores to simulate the formation of brine. An N_2_ molecule box was placed on the left side of the silica structures in order to create a pressure differential in the initial model. A vacuum slab was placed on the right side of the silica structures. A rigid graphene sheet was placed on the left side of the N_2_ flooding and the right side of the vacuum slab as a rigid piston (Figure 1). For the system, the pores were uniformly filled with water/salt at the initial moment. A 1 ns equilibrium MD simulation with the NVT ensemble was conducted to achieve an equilibrium state [19].

The well-known 12–6 Lennard–Jones potential 4ε[(σ/r)^12^ − (σ/r)^6^] [20] was used to calculate the van der Waals interaction, where r represents the distance between two atoms. The cutoff distance was set to 11 Å. The rigid SPC/E model [21] was used for modeling water molecules with the SHAKE algorithm [22], while the Lennard–Jones potential was adopted to model the neutral N_2_ [23]. The silica surface was modeled by the CLAYFF [24] force field. All MD simulations were performed in the NVT ensemble (the number of molecules, volume, and temperature were constant) with a time step of 1 fs [25]. The temperature, kept at 298 K, was controlled by a Nosé–Hoover thermostat [26], and every 1000 steps, a frame was set for data analysis. LAMMPS software [27] was employed, and total simulation was conducted for 1 ns. The application of the periodic boundary condition was done in all directions [28]. The desired transmembrane pressure in the experiments was applied to the feed piston to push the water/salt across the membrane.

## 3. Discussion

During the injection process, N_2_ passed through the pores of SiO_2_ with breakthrough pressures of 35.83, 14.00, 11.18, 5.77, and 0.50 MPa, which were selected to put the interlayer porosities of 4.29%, 4.73%, 5.17%, 7.22%, and 11.38% in the displacement systems, according to the experimental data (offered by the Research Institute of Exploration and Development, PetroChina Xinjiang Oilfield Company). Breakthrough pressures were applied to the rigid graphene sheet. 

### 3.1. Displacement of Water/Salt by N_2_

**Figure 2:**
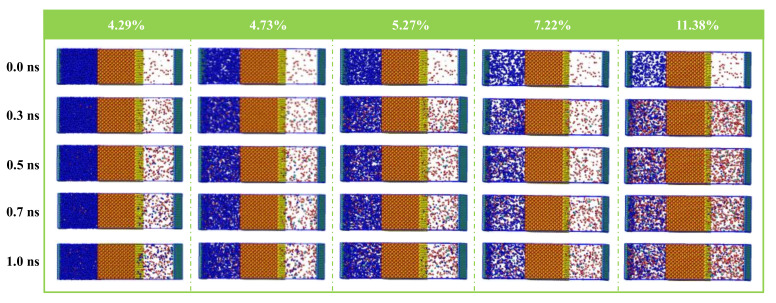
Side views for the evolution of N_2_ flooding from 0 to 1 ns.

Figure 2 presents the process snapshots of N_2_ injection in the porosities of 4.29%, 4.73%, 5.17%, 7.22%, and 11.38%. For a certain porosity, H_2_O, Na^+^, and Cl^−^ ions were displaced after the injection of N_2_, and the displacement degree of H_2_O, Na^+^, and Cl^−^ gradually increased with time. Taking the structure with a porosity of 4.29% as an example, all N_2_ molecules were gathered on the left side of the SiO_2_ initially. After 0.3 ns, some N_2_ molecules passed through the SiO_2_ phase, and meanwhile, some H_2_O, Na^+^, and Cl^−^ were displaced into the vacuum phase and the N_2_ phase. With an increase in simulation time, more N_2_, H_2_O, Na^+^, and Cl^−^ passed through the SiO_2_ phase until the system reached equilibrium at 0.7 ns. In comparison, the larger porosities led to more evident displacement phenomena after a period of time. When the porosity was 11.38%, almost all the H_2_O dissolved into the N_2_ phase. Detailed observations indicated that after injecting N_2_, all components were in dynamic equilibrium within the interlayer after 1 ns.

**Figure 3:**
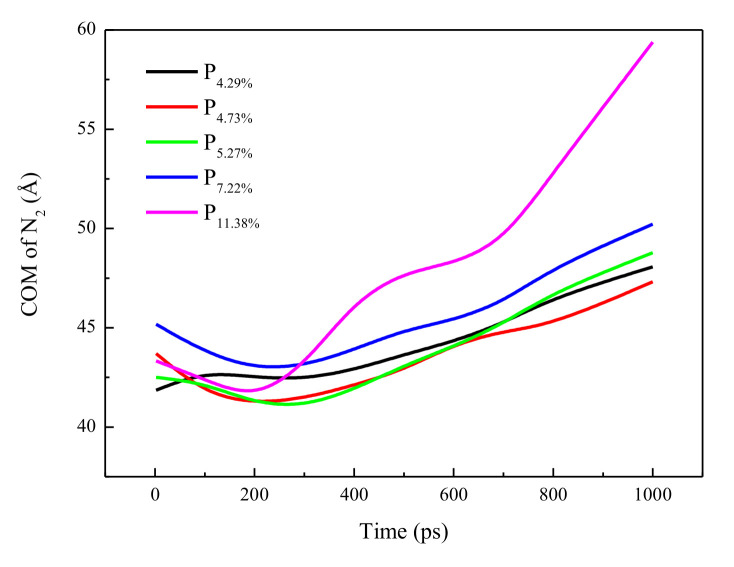
The location of the center of mass of N_2_.

To analyze the displacement process, the center of mass (COM) of N_2_ was explored, as shown in Figure 3. For certain interlayer structures, the COM of N_2_ moved toward the right of the interlayer structures. After 0.3 ns, the COM of N_2_ moved −0.65 Å, −2.52 Å, −1.30 Å, −2.24 Å, and −1.93 Å for P4.29%, P4.73%, P5.27%, P7.22%, and P11.38%, respectively. In other words, the COM of N_2_ moved far toward the left of the interlayer structures at the beginning of the simulation, except for the structure with a porosity of 4.29%. This was attributed to the achievement of an equilibrium state. In the next 0.7 ns, the COM of N_2_ moved toward the right of the interlayer structures with moving distances of 5.66 Å, 5.87 Å, 7.74 Å, 7.14 Å, and 16.29 Å for P4.29%, P4.73%, P5.27%, P7.22%, and P11.38%, respectively. After the same simulation time, large porosity led to an increase in the movement distance of the COM, which was consistent with the process snapshot analysis. For P11.38%, the large pore size led to more gas molecules passing through interlayer structures, resulting in a faster passing process than other structures. Therefore, the curve of P11.38% shows an extremum and a large slope.

### 3.2. Movability Evaluation

Permeance is an indispensable indicator to determine the displacement efficiency of N_2_ over water/salt [3]. Table 1 shows the permeances of N_2_ passing through SiO_2_ with five interlayer structures with different porosities. As the porosity increased, the permeance of N_2_ gradually increased in the sequence of P4.29% < P4.73% < P5.27% < P7.22% < P11.38%. A large pore size results in a small barrier, resulting in a small N_2_ energy repulsion. Moreover, the weak interaction guarantees that the gas molecules pass through the pores of SiO_2_ rapidly.

**Figure 4:**
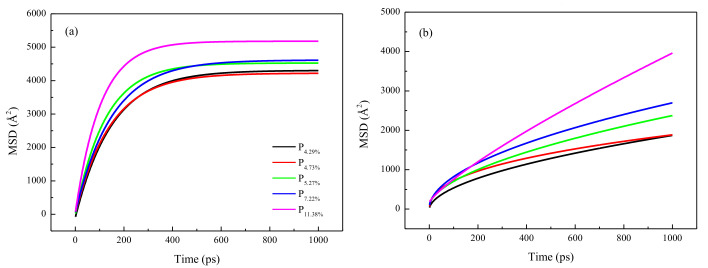
The mean squared displacement of H_2_O (**a**) and N_2_ (**b**).

In order to evaluate the diffusion behavior and movability of N_2_ and H_2_O molecules, mean squared displacement (MSD) [29,30] was calculated:

(1) $$\mathrm{MSD}={\left|R_{i}\left(t\right){-R}_{i}\left(0\right)\right|}^{2}$$

where $R_{i}\left(t\right)$ represents the position of i atom at t time, and $R_{i}\left(0\right)$ represents the initial position. Each pore was divided into eleven layered blocks, and MSD was calculated for each structure. Previous studies confirmed that the gas diffusion rate was positively correlated with the slope of the MSD profile. As exhibited in Figure 4a, on the whole, the MSD of H_2_O along the *z*-axis in the five interlayers increased sharply at the beginning of the simulation (0–200 ps). The slope of the MSD followed the sequence of P11.38% > P7.22% > P5.27% > P4.73% > P4.29%. In particular, the structure with a porosity of 11.38% showed the fastest gas diffusion because the large pore size enhanced the H_2_O diffusion rate. After the initial increase in the MSD, the slope of the MSD flattened out to a constant value because of the equilibrium state of the system. During this period (200–1000 ps), the equilibrated MSD of H_2_O followed the sequence of P11.38% > P7.22% > P5.27% > P4.73% > P4.29%. The trend of the MSD curve agreed well with the physical characteristics and porosity of the pores.

Figure 4b delineates the MSD of N_2_ along the *z*-axis in the nanopores of SiO_2_ with an increase in time. Larger porosity significantly boosted the diffusion capacity of N_2_. During the simulation, the porosity of the interlayer structures increased from P4.29% to P4.73%, P5.27%, P7.22%, and P11.38%, and the equilibrated MSD of N_2_ increased to 0.17%, 33.44%, 45.11%, and 117.61%. This result confirms that the MSD of N_2_ is more significantly enhanced by the large porosity of P11.38% than by those of other structures. Conversely, the MSD of H_2_O is more affected by porosity. In short, increasing porosity can enhance the MSD of H_2_O more than that of N_2_ at both early and late stages of the simulation, particularly for the large P11.38%. Therefore, it can be envisioned that large porosity can greatly promote the diffusion of N_2_ in SiO_2_ for the displacement of water and salt. Higher mobility of the gas molecules in the middle of the interlayer was observed for structures with large porosity, and the moving ability near the small silica surface was poor. However, over time, the overall effect of the H_2_O slug flooding became more severe than that of N_2_.

### 3.3. Distribution of H_2_O, N_2_, and Salt in Interlayer Structure

**Figure 5:**
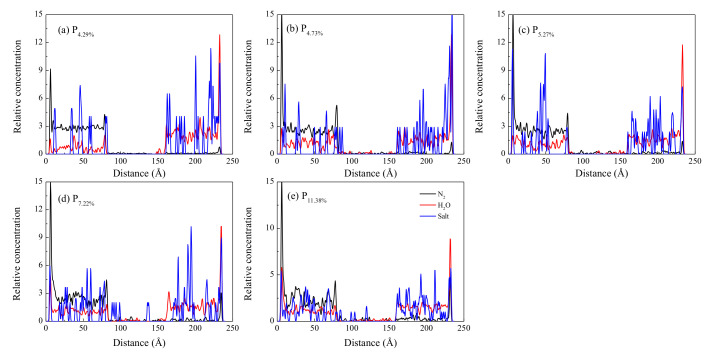
Relative concentration distribution of N_2_, H_2_O, and Salt along z-axis.

In order to evaluate the distribution of the H_2_O, N_2_, and salt in the Z direction visually, the relative concentration profiles over 1 ns were calculated, as shown in Figure 5. On the *z*-axis, the range of 0–71 Å represents the N_2_ phase, the range of 75–165 Å represents the porous SiO_2_ phase, and the range of 170–240 Å is the vacuum phase. The rigid graphene sheet was placed on the left side of the N_2_ flooding and the right side of the vacuum slab as a rigid piston. Initially, most of the H_2_O, Na^+^, and Cl^−^ was in the pores of the SiO_2_. After 100 ps, the density difference of the N_2_/H_2_O/salt at the SiO_2_ interface gradually diminished, while a substantial change in the density of the N_2_/H_2_O/salt inside and outside the pores of the SiO_2_ took place. The small pore size of the SiO_2_ with a porosity of 4.29% limited the number of molecules in the free phase, resulting in almost no N_2_ molecules left in the pores. The lower porosity led to a smaller volume to fill. And less N_2_ in the free phase. However, some H_2_O with dipole moment, Na^+^, and Cl^−^ ions existed in the pores of the SiO_2_, which is explained by the larger porosity. On the whole, some N_2_ molecules can pass through the pores of the SiO_2_ into the vacuum phase in all five systems under the breakthrough pressure. However, most N_2_ molecules remain in the pristine N_2_ phase. The high peak in the range of 0–10 Å was attributed to N_2_ adsorption in the graphene sheet surface, and another peak in the range of 75–80 Å was attributed to N_2_ adsorption in the SiO_2_ surface, while the remaining N_2_ molecules were uniformly distributed. The H_2_O and salt diffused from the pores of the SiO_2_ into the N_2_ and vacuum phase, and the number of H_2_O, Na^+^, and Cl^−^ ions in the N_2_ phase was smaller than that in the vacuum phase. The results show that N_2_ slugs can effectively displace the saline solution in the pores of the SiO_2_ into the vacuum phase.

### 3.4. Interaction between N_2_ and H_2_O

**Figure 6:**
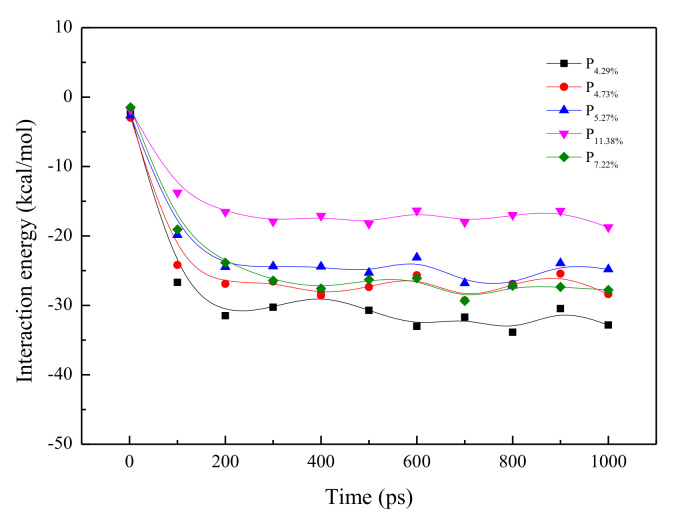
The interaction relationship between N_2_ and H_2_O with the increase in time.

Figure 6 presents the interaction energy between N_2_ and H_2_O with simulation time. The interaction between N_2_ and H_2_O was negative during the whole process, and large negative values indicate a strong interaction between N_2_ and H_2_O. Overall, as the time increased, the interaction energy between N_2_ and H_2_O initially increased rapidly and then plateaued to a constant. From 0 ps to 200 ps, the interaction energy increased from 0 to −31.48, −26.89, −24.56, −23.85, and −16.54 kcal/mol for P4.29%, P4.73%, P5.27%, P7.22%, and P11.38%, respectively. The interaction energy followed the sequence P11.38% < P7.22% < P5.27% < P4.73% < P4.29%, which agreed with the porosity of SiO_2_. This result implies that few N_2_ and H_2_O molecules are initially in contact with each other at the interface between N_2_ slugs and SiO_2_, and more N_2_ molecules were in contact with H_2_O as time increased until the system reaches equilibrium. The interaction in P4.29% was much higher than that in other structures because of the strengthened interaction between N_2_ and H_2_O by the small pore size. During the simulation time of 200–1000 ps, N_2_ molecules passed through the pores of the SiO_2_, and H_2_O molecules diffused into the N_2_ box as well as the vacuum phase. 

### 3.5. The Relationship between the Parameters

**Figure 7:**
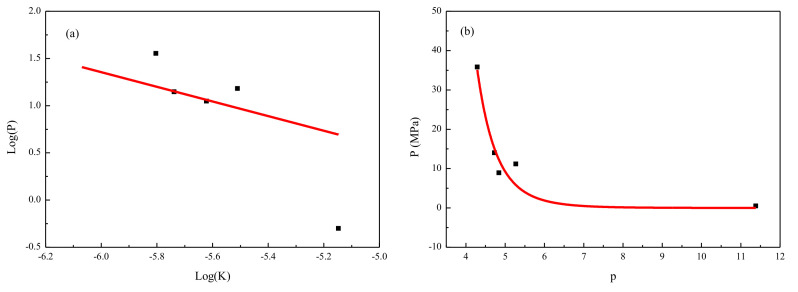
(**a**) The relationship between permeability and breakthrough pressure; (**b**) the relationship between permeability and porosity.

To investigate the relationship between permeability, porosity, and breakthrough pressure, the proper function fittings are discussed. Figure 7a shows the relationship between the permeability of N_2_ and the breakthrough pressure of N_2_. After logarithm fetching on the permeability of N_2_ (Log(K)), a linear relationship with the logarithm fetch breakthrough pressure of N_2_ (Log(P)) was given. The fitting formula is Log(P) = −0.775 × Log(K) − 3.298, which is in agreement with the experimental results of Log(P) = −0.658 × Log(K) − 0.557, which was called by log–log analysis. The same order of magnitude for the line slope and the experimental results shows the rationality of the model and accuracy of the calculation parameters. Furthermore, the large permeability can be obtained by adjusting the breakthrough pressure according to the fitting formula. In addition, the intersection profile of the porosity and gas injection breakout pressure was fitted, indicating an exponential relationship between them. The large porosity corresponds to smaller breakout pressure according to the fitting formula. In order to show the relationship between porosity (P) and permeability (p), their fitting profile is shown in Figure 7b. The fitting formula is P = 1.13 × 10^7^ ×${\;e}^{-8.71p}$, which is a typical exponential relationship. In the process of oil and gas exploitation, the character of the pore structure is first acquired, the gas injection breakout pressure is calculated next by the intersection profile, and last, the permeability is predicted by log–log analysis in theoretical calculations.

## 4. Conclusions

The penetration and displacement behavior of molecules in interlayers with different porosities were analyzed using molecular dynamics simulations. The results show that interlayers with different pore sizes and porosities can strongly affect the distribution, diffusion, and displacement processes. The MSD and gas distribution show that N_2_ can pass through the pores of interlayer structures and displace the H_2_O, Na^+^, and Cl^−^ in the pore space into the vacuum phase. This shows that high porosity can result in high-efficiency diffusion of N_2_, and high porosity in porous interlayers benefit the displacement of H_2_O, Na^+^, and Cl^−^. Interaction analysis between N_2_ and H_2_O confirms that a strong interaction between N_2_ and water can overcome the interaction between water and the pore wall to render rapid displacement of H_2_O, Na^+^, and Cl^−^. A log–log analysis and an intersection profile were presented to theoretically predict the relationship between permeability, porosity, and breakthrough pressure. 

## Figures and Tables

**Table 1:** The permeances of N_2_ passing through the five interlayers.

Porosity	4.29%	4.73%	5.27%	7.22%	11.38%
Permeability	1.57 × 10^−6^	1.83 × 10^−6^	2.39 × 10^−6^	3.09 × 10^−6^	7.12 × 10^−6^

## Data Availability

No new data were created or analyzed in this study. Data sharing is not applicable to this article.
